# Comparative Evaluation of Soluble and Insoluble-Bound Phenolics and Antioxidant Activity of Two Chinese Mistletoes

**DOI:** 10.3390/molecules23020359

**Published:** 2018-02-08

**Authors:** Qing Li, Shihua Yang, Yongqiang Li, Xiaofeng Xue, Yonghua Huang, Hengguo Luo, Yiming Zhang, Zhichao Lu

**Affiliations:** 1 College of Food Science and Technology, Yunnan Agricultural University, Kunming 650201, China; 13211674852@163.com (Q.L.); hyh209920@163.com (Y.H.); l15559818968@163.com (H.L.); zym718zhangym@163.com (Y.Z.); luzhichao1s@163.com (Z.L.); 2 College of Foreign Languages, Yunnan Agricultural University, Kunming 650201, China; yanglifang815@163.com; 3 Institute of Apicultural Research, Chinese Academy of Agricultural Sciences, Beijing 100093, China

**Keywords:** Chinese mistletoes, phenolics, phenolic contents, antioxidant activity

## Abstract

Mistletoes are used medicinally in order to treat various human illnesses. Few studies have reported on the phenolic content and antioxidant properties of Chinese mistletoes (CMs). In this work, the total phenolic content (TPC), total flavonoid content (TFC), and antioxidant activities of soluble and insoluble-bound phenolic extracts from CMs hosted by *Camellia assamica* (Mast.) Chang (CMC) and *Pyrus*, *i*, *f.* (CMP) were compared. Phenolic compounds in CMC and CMP were identified and quantified using high-performance liquid chromatography (HPLC). The results indicated that the TPC of soluble phenolic extracts was higher than insoluble-bound phenolic counterparts in both CMC and CMP. In addition, the TPC of soluble, insoluble-bound and total phenolic fractions (9.91 ± 0.23, 4.59 ± 0.27 and 14.50 ± 0.35 μmol ferulic acid equivalents per gram (FAE/g) dry sample) extracted from CMP were higher than those extracted from CMC. The soluble phenolic extracts in CMP showed higher antioxidant activities than those in CMC. Eighteen phenolic compounds from soluble and insoluble-bound phenolic extracts from the CMs were identified and quantified by HPLC. This study indicates that CMC and CMP, especially the latter, could be sources of antioxidants in human health care.

## 1. Introduction

Mistletoes belonging to the order Santalales, which comprises Santalaceae, Loranthaceae, and Misodendraceae, are semi-parasites that grow on the Theaceae, Rosaceae, Moraceae and Leguminosae families [1]. Most are distributed in Southern and Central Asia, North-Western Africa, Europe and Eastern Australia [2,3,4]. Since ancient times, mistletoes have been applied as traditional medicines for the treatment of cancer and some chronic diseases due to their antioxidant and anti-inflammatory activities [3,5,6,7].

The main phytoconstituents, including proteins, carbohydrates, flavonoids, glycosides, phenolic compounds, tannins and triterpenes, have been identified in mistletoes by their physicochemical properties and spectral analysis [8,9,10]. In addition, several studies have revealed that mistletoes possess moderate antioxidant capacity due to the presence of phenolic compounds [9,10,11]. *Viscum articulatum* Burm. f. hosted by *Camellia assamica* (Mast.) Chang (CMC) and *Viscum liquidambaricolum* Hayata parasitic on *Pyrus*, *i*, *f.* (CMP) are two native Chinese mistletoes (CMs). CMC has been commonly used in traditional Chinese medicine for the treatment of hemorrhage, pleurisy, gout, heart disease, epilepsy, arthritis, and hypertension [12]. Previous investigations of CMC have revealed that phenolic glycosides, flavanone glycosides, triterpenoids, organic acids and flavonoids are the major secondary metabolites of this plant [13,14]. A few studies have focused on the phenolic composition and antioxidant activities of soluble phenolic compounds in CMC. However, there has been little research into insoluble-bound phenolic compounds in CMC. Moreover, the phenolic composition and antioxidant activities of CMP are unclear.

Phenolic compounds produced during secondary metabolism are characterized by some phenolic hydroxyl groups in the molecules [15]. These can be divided into several groups, including phenolic acids, flavonoids, stilbenes and lignans, based on the chemical structure of the phenolic compounds [16]. According to their solubility features, phenolics are separated into soluble and insoluble-bound fractions [17,18,19]. Insoluble-bound phenolics are covalently bound to the cell–wall matrix, including cellulose, arabinoxylans and proteins by ester, ether and carbon–carbon bonds [18]. Organic solvent is used to extract the soluble phenolic compounds, whereas acidic, alkaline or enzymatic hydrolysis are used to release insoluble-bound phenolics [19]. Insoluble-bound phenolics may be slowly and continuously released in the human gastrointestinal tract and during colonic fermentation, which can improve bioaccessibility and potential bioavailability and exert high bioactivity on tissues and cells for a long time [18,20]. However, most studies reported in the literature have ignored insoluble-bound phenolic compounds, and hence have underestimated their phenolic compound content and activities. Phenolic compounds are major sources of dietary antioxidants in the plants [21]. Antioxidants have beneficial health aspects, preventing and scavenging free radicals by means of donating hydrogen atoms to a free radical in order to protect biomolecules, such as proteins, lipids, carbohydrates and DNA [20,22,23], and to alleviate chronic diseases and degenerative ailments [24]. Thus, the beneficial effects of mistletoes may be attributed to their phenolic compounds [21].

The objectives of this study were to quantify the total phenolic and flavonoid contents of soluble and insoluble-bound phenolic extracts and to assess the antioxidant activities of CMC and CMP. The phenolic compounds in these two Chinese mistletoes were identified and quantified by high-performance liquid chromatography (HPLC). Moreover, the content, compositions and antioxidant activities of phenolic extracts in CMC and CMP were compared.

## 2. Results and Discussion

### 2.1. Total Phenolic Content (TPC) and Total Flavonoid Content (TFC)

**Figure 1:**
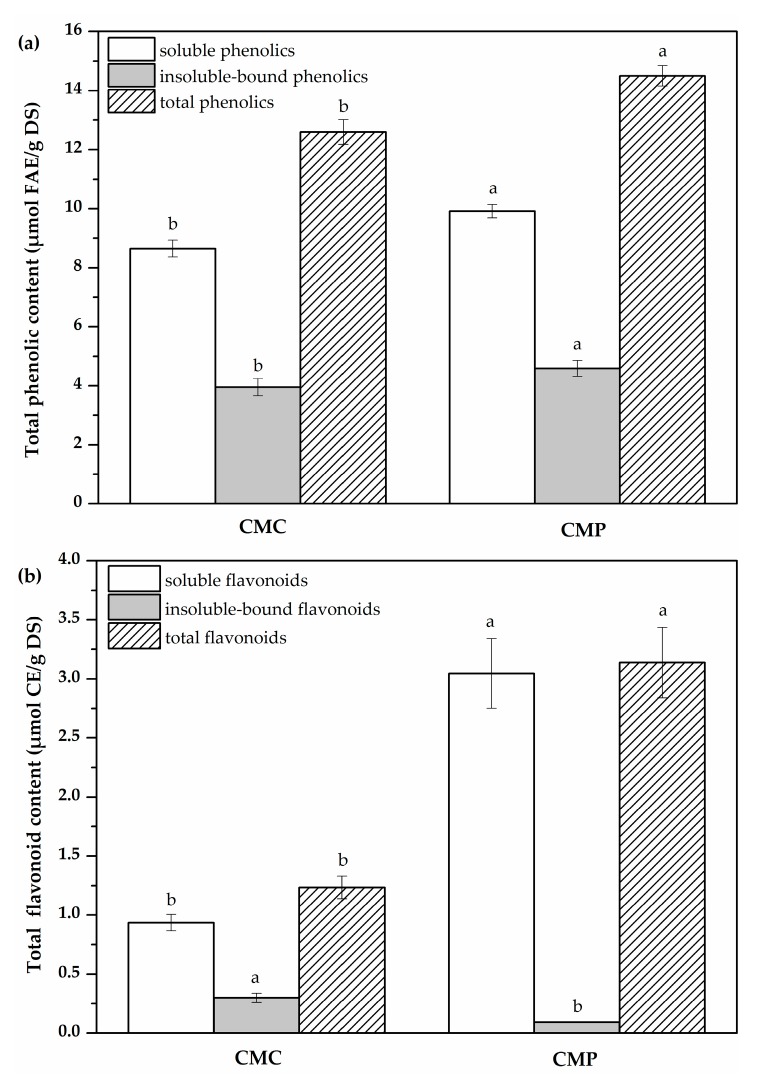
Total phenolic content (**a**) and flavonoid content (**b**) of soluble and insoluble-bound phenolic extracts in the two Chinese mistletoes (CMs). Different letters in each category (soluble, insoluble-bound, and total phenolics) are significantly different (*p* < 0.05). CMC, the Chinese mistletoes hosted by *Camellia assamica* (Mast.) Chang; CMP, the Chinese mistletoes hosted by *Pyrus*, *i*, *f*; FAE, ferulic acid equivalents; CE, catechin equivalents; DS, dry sample.

Phenolics are the predominant group of phytochemical compounds, and are widely distributed in flowers, fruits, seeds, roots, stems, leaves of various plants and medicinal herbs [3,25,26]. Figure 1 presents the TPC and TFC of soluble and insoluble-bound phenolic extracts of CMC and CMP. The TPC of soluble, insoluble-bound and total phenolic extracts in CMs ranged from 8.65–9.91 μmol FAE/g DS, 3.95–4.59 μmol FAE/g DS and 12.59–14.50 μmol FAE/g DS, respectively. The TPC of soluble phenolic extracts, either in CMC or in CMP, was significantly higher (*p* < 0.05) than that of insoluble-bound phenolic content. Similar results have been obtained for the TPC of millet, barley, onion and the different parts of *Castanea crenata* [22,26,27]. However, in contrast to the results obtained in the present works, some researchers have found that the TPC of soluble phenolic extracts was lower than insoluble-bound phenolics [28,29], which may be due to the differences of bond strength between phenolic compounds and cell-wall matrix. Moreover, the TPC of soluble, insoluble-bound and total phenolic in extracts of CMP were significantly higher than those in CMC (*p* < 0.05).

Flavonoids are phenolic compounds that exhibit various biological activities, such as anti-cancer, anti-allergenic, anti-viral, anti-inflammatory effects, vasodilating actions and gastroprotective properties, as well as having superior antioxidant activities [30]. The TFC of soluble, insoluble-bound and total flavonoids extracts in CMs ranged from 0.93–3.05 μmol CE/g DS, 0.10–0.30 μmol CE/g DS and 1.23–3.14 μmol CE/g DS, respectively. The TFC of the soluble and insoluble-bound flavonoids extracts in CMC and CMP exhibited a similar trend to that of TPC. The results showed that the TFC of the soluble flavonoids fractions in CMs was higher than corresponding insoluble-bound flavonoids. Similar results have been reported in previous studies [22,27]. The TFC of soluble flavonoid extracts in CMP was significantly higher (*p* < 0.05) than that in CMC. However, the TFC of insoluble-bound flavonoids extracts in CMC was significantly higher than that in CMP (*p* < 0.05). This discrepancy may be attributed to majority flavonoids that could bind the proteins and polysaccharides through ether and ester bonds in the CMC [18].

The results of our studies demonstrated that the TPC and TFC of both soluble and insoluble phenolic extracts were different in CMC and CMP. In addition, the TPC and TFC of total soluble phenolics in CMP were higher than those of in CMC.

### 2.2. Antioxidant Activities In Vitro

The antioxidant capacities of phenolic extracts can be measured in several ways. In this study, four different and complementary methods (ferric reducing antioxidant power (FRAP), hydrogen peroxide scavenging activity (HPSA), DPPH radical scavenging activity (DRSA) and Trolox equivalent antioxidant capacity (TEAC)) were used to describe more fully the antioxidant capacities in vitro. Although these assays are of limited use in predicting health benefits in humans, and extrapolation to an in vivo situation is not possible, they may still be valuable as a screening method for predicting the antioxidant activities of phenolic compounds [31,32].

The antioxidant activities of the soluble and insoluble-bound phenolic compounds extracted in CMC and CMP were detected (Table 1). It has been reported that there is a correlation between antioxidant activities and total phenolic content in many plants [9,15,25,26,33,34,35]. The FRAP of soluble and insoluble-bound phenolic extracts in CMs ranged from 42.25–44.76 μmol FE/g DS and 8.07–10.31 μmol FE/g DS. The HPSA of two phenolic extracts in CMs were in the range of 1429.34–1431.87 μmol FAE/g DS and 1383.79–1231.67 μmol FAE/g DS. The DRSA and TEAC of soluble phenolic extracts in CMs ranged from 2.19–2.51 μmol FAE/g DS and 81.03–84.92 μmol TE/g DS, and those of insoluble-bound phenolics ranged from 1.51–1.83 μmol FAE/g DS and 5.78–1.40 μmol TE/g DS. The FRAP, HPSA, DRSA and TEAC of the soluble phenolic extracts in CMs were significantly higher than their insoluble-bound phenolic counterparts (*p* < 0.05). The same trends of antioxidant activities were observed in millet, barley and onion [22,27]. In addition, soluble phenolic extracts in CMP had higher antioxidant capacities in terms of FRAP, HPSA, DRSA and TEAC than those in CMC. However, it was found that insoluble-bound phenolic fractions in CMP had significantly higher FRAP and DRSA values than those in CMC (*p* < 0.05). Therefore, our results showed that CMP with higher phenolic and flavonoid contents had stronger antioxidant activities than CMC. Thus, the two CMs might be used as food additives because their phenolic extracts had good antioxidative and radical-scavenging activities.

### 2.3. Identification and Quantification of the Two Chinese Mistletoe (CM) Extracts by High-Performance Liquid Chromatography (HPLC)

**Figure 2:**
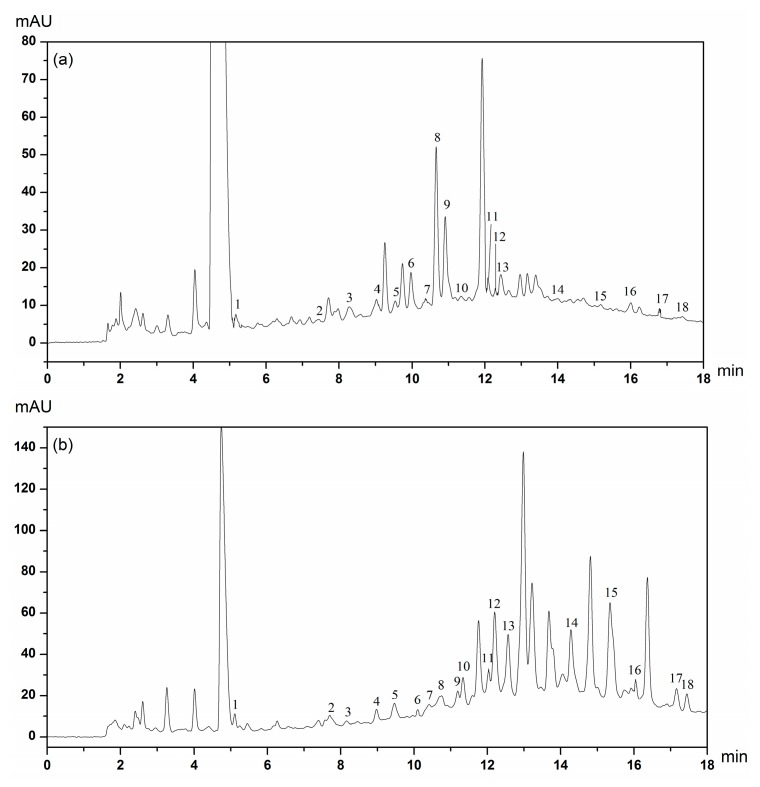
HPLC chromatograms of soluble phenolic extracts of the Chinese mistletoes hosted by *Camellia assamica* (Mast.) Chang (CMC) (**a**) and *Pyrus*, *i*, *f*. (CMP) (**b**). The identified compounds: 1, Gallic acid; 2, Protocatechuic acid; 3, Catechin hydrate; 4, Chlorogenic acid; 5, *p*-Hydroxybenzoic acid; 6, Epicatechin; 7, Caffeic acid; 8, Vanillic acid; 9, Syringic acid; 10 Vanillin; 11, (−)-Epigallocatechin; 12, *p*-Coumaric acid; 13, Ferulic acid; 14, Myricetin; 15, Quercetin; 16, *trans*-Cinnamic acid; 17, Kaempferol; 18, Apigenin.

**Figure 3:**
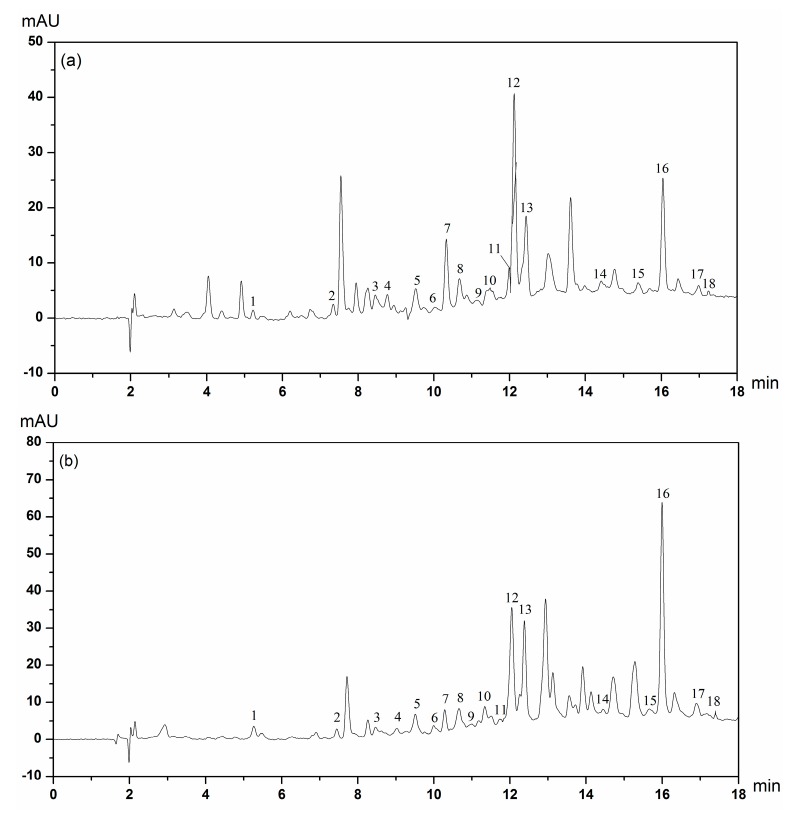
HPLC chromatograms of insoluble-bound phenolic extracts of the Chinese mistletoes hosted by *Camellia assamica* (Mast.) Chang (CMC) (**a**) and *Pyrus*, *i*, *f*. (CMP) (**b**). The identified compounds: 1, Gallic acid; 2, Protocatechuic acid; 3, Catechin hydrate; 4, Chlorogenic acid; 5, *p*-Hydroxybenzoic acid; 6, Epicatechin; 7, Caffeic acid; 8, Vanillic acid; 9, Syringic acid; 10 Vanillin; 11, (−)-Epigallocatechin; 12, *p*-Coumaric acid; 13, Ferulic acid; 14, Myricetin; 15, Quercetin; 16, *trans*-Cinnamic acid; 17, Kaempferol; 18, Apigenin.

The HPLC chromatograms of soluble and insoluble-bound phenolic compounds in CMC and CMP extracts are presented in Figure 2 and Figure 3. The main classes of phenolic compounds identified in CMC and CMP were hydroxybenzoic acids, hydroxycinnamic acids and flavonoids.

The phenolic compounds of soluble phenolic extracts are shown in Table 2. Several hydroxybenzoic acids, including gallic acid, protocatechuic acid, *p*-hydroxybenzoic acid, vanillic acid, syringic acid and vanillin, were identified in CMC and CMP by comparison of their retention time (RT) with those of the available standards (Figure S1). Our results showed that the RT of phenolic compounds of hydroxybenzoic acids in CMP were 1.95–5.88 times higher than those in CMC, except for vanillic acid and syringic acid. The major hydroxycinnamic acids identified were chlorogenic acid, caffeic acid, *p*-coumaric acid, ferulic acid and *trans*-cinnamic acid. In general, the RT of caffeic acid, *p*-coumaric acid and *trans*-cinnamic acid in CMP were 1.12, 9.64 and 2.54 times higher than those in CMC. Flavonoids, namely catechin hydrate, epicatechin, (−)-epigallocatechin, myricetin, quercetin, kaempferol and apigenin were determined. Flavonoids are a large family of compounds in plants [30]. The content of all six phenolic compounds in CMP was higher than those in CMC. In addition, our results showed that myricetin (2209.79 ± 1476.96 μg/g) and epicatechin (238.18 ± 79.30 μg/g) were the most abundant in CMP and CMC, respectively. Previous research has shown that quercetin was the most abundant among five Polish *Viscum album* [9]. The difference between Polish *Viscum album* and CMs may be due to the varieties and the growth conditions of the plants. In addition, flavonoids constituted a substantial content of total phenolic compounds and individually contributed to 24.55% and 60.94% of the content in CMC and CMP, respectively. Vanillic acid (1325.77 ± 23.34 μg/g) and myricetin (2209.79 ± 1476.96 μg/g) might be characteristic phenolic compounds in CMC and CMP, respectively, due to their high contents. Most of the eighteen phenolic compounds were also found in different mistletoes [9,14,36]. Furthermore, myricetin had been recognized as a source that could limit type 2 diabetes mellitus [37]. Hence, the phenolic compounds of CMs have potential to be researched further. The insoluble-bound phenolic profiles are given in Table 3. Eighteen phenolic compounds subdivided into hydroxybenzoic acids, hydroxycinnamic acid and flavonoids, were also identified and quantified by HPLC. Moreover, *p*-coumaric acid (206.97 ± 21.39 μg/g) and (−)-epigallocatechin (223.32 ± 24.87 μg/g) might be characteristic phenolic compounds in insoluble-bound phenolic extracts of CMC and CMP, respectively.

## 3. Materials and Methods

### 3.1. Materials and Chemical Reagent

*Viscum articulatum* Burm. f. (CMC) and *Viscum liquidambaricolum* Hayata (CMP) were purchased from Pu’er, Yunnan province, China in 2016. Standard phenolic compounds (gallic acid, protocatechuic acid, chlorogenic acid, caffeic acid, *p*-coumaric acid, (−)-epigallocatechin, myricetin, kaempferol, apigenin, epicatechin, quercetin, vanillic acid, syringic acid, *trans*-cinnamic acid, catechin, ferulic acid, *p*-hydroxybenzoic acid and vanillin) were purchased from Beijing Beina Chuanglian Biotechnology Institute (Beijing, China). Folin-Ciocalteu phenol reagent, 6-hydroxy-2,5,7,8-tetramethylchroman-2-carboxylic acid (Trolox), 2,2-diphenyl-1-picrylhydrazyl (DPPH) were obtained from Sigma-Aldrich (St. Louis, MO, USA). 2,4,6-Tripyridyl-s-triazine (TPTZ), 2,2′-azinobis (3-ethylbenzothiazoline-6-sulfonic acid) (ABTS), trichloroacetic acid, ferric chloride, ascorbic acid, ferrous sulfate, potassium ferricyanide, sodium phosphate dibasic, sodium phosphate monobasic dihydrate, H_2_O_2_, aluminum chloride and potassium persulfate were purchased from Aladdin Industrial Corporation (Shanghai, China). HPLC-grade methanol and formic acid were purchased from Merck (Darmstadt, Germany). All chemicals used in the experiments were of analytical grade.

### 3.2. Separation of Phenolic Compounds

The soluble and insoluble-bound phenolic compounds from the two CMs were prepared using the methods reported in literature [20,38] with slight modifications. The mistletoes were ground to a fine powder with a Wiley mill (1029-A, Yoshida Seisakusho Co., Tokyo, Japan) for herbal medicine, and screened through a 50-mesh sieve. After the powder was freeze-dried with a vacuum freeze dryer (LGJ-12, Zhengzhou Nanbei Instrument Equipment Co., Ltd., Zhengzhou, China), 40 mL of 70% (*v*/*v*) acetone was added to 2.0 g of the dried powder, and then samples were shaken in an ultrasonic bath (SB-3200D, Ningbo Xinzhi biological Polytron Technologies Inc. 300 W, Ningbo, China) at room temperature for 15 min. The mixture was centrifuged at 4000× *g* for 10 min at 4 °C (TGL20M, Hunan Xiang Li Scientific Instrument Co., Ltd. Hunan, China). The upper layer was collected, and the extractions were repeated twice. The supernatants were combined and evaporated under reduced pressure at 30 °C (RE-52 AA, Shanghai Yarong biochemical instrument factory, Shanghai, China). This extract solution was analyzed as soluble phenolic extract.

The residues were used to extract the insoluble-bound phenolics. The samples were subsequently hydrolyzed with 40 mL NaOH (4 mol/L) at ambient temperature under nitrogen gas for 4 h. The resultant hydrolysate was acidified to pH 2 using HCl (6 mol/L) and then centrifuged at 4000× *g* for 10 min at 4 °C. The supernatants were combined and extracted 3 times with an equal volume of diethyl ether and ethyl acetate at 1:1 (*v*/*v*), and then evaporated under reduced pressure (30 °C). The insoluble-bound phenolic compounds were obtained. All samples were dissolved in 25 mL of HPLC grade methanol, and stored at −20 °C under nitrogen gas and covered with aluminum foil until used.

### 3.3. Determination of TPC

The TPC was determined using Folin–Ciocalteu phenol reagent, followed by the Chandrasekara and Shahidi [20] and Singleton and Rossi [39] methods, with slight modifications. Briefly, 500 μL of each phenolic extract was added to 0.5 mL Folin–Ciocalteu phenol reagent (2 mol/L) and 1 mL of saturated sodium carbonate (75 g/L). After adding distilled water (to a total volume of 10 mL) and thorough mixing, the mixture was allowed to stand at ambient temperature in the dark for 35 min and centrifuged at 4000× *g* for 10 min at 4 °C. The absorbance of this solution versus a prepared blank was measured at 760 nm. The content of total phenolics in each sample was determined using a standard curve prepared for ferulic acid and expressed as micromoles (μmol) of ferulic acid equivalents (FAE) per gram of dry sample (DS) (μmol FAE/g DS).

### 3.4. Determination of TFC

The TFC was measured using the aluminum chloride colorimetric method as described by Kern et al. [40] and Chandrasekara and Shahidi [41], with slight modifications. Briefly, 2 mL of each phenolic extract was added to 4 mL of distilled water and 0.3 mL of 5% NaNO_2_. Five minutes later, 0.3 mL of 10% AlCl_3_ was added to the reaction mixture and allowed to react for 1 min. Finally, 2 mL of 1 mol/L NaOH and 1.4 mL of distilled water were added and mixed as quickly as possible. The mixture was centrifuged at 4000× *g* for 5 min at 4 °C after incubation at ambient temperature in the dark for 15 min. The absorbance of this solution versus a prepared blank was measured at 510 nm. Catechin was used as a reference standard, and the results were expressed as μmol of catechin equivalents (CE) per gram of dry sample (μmol CE/g DS).

### 3.5. Determination of Ferric Reducing Antioxidant Power (FRAP)

The FRAP method was based on the procedure described by Benzie and Strain [42] and Villanueva-Carvajal et al. [43], with slight modifications. Briefly, the FRAP working solution was prepared from acetate buffer (300 mmol/L, pH 3.6), FeCl_3_ solution (20 mmol/L), and 2,4,6-tripyridyl-s-triazine (10 mmol/L) in a volume ratio of 1:1:1. The phenolic extracts (100 μL) were mixed with 3 mL of the FRAP working solution and incubated at 37 °C in the dark for 4 min. The absorbance of the solution was measured at 539 nm. Ferrous sulfate was used as a reference standard, and the FRAP was expressed as μmol of Fe^2+^ equivalents (FE) per gram of dry sample (μmol FE/g DS).

### 3.6. Determination of H_2_O_2_ Scavenging Activity (HPSA)

The HPSA was measured by using the method described by Wettasinghe and Shahidi [44] and Chandrasekara et al. [41], with slight modifications. Briefly, the phenolic extracts (600 μL) mixed with 0.9 mL of H_2_O_2_ (40 mmol/L) and 1.5 mL of sodium phosphate buffer (45 mmol/L, pH 7.4), and the resulting solution was left to stand at 30 °C in the dark for 40 min. Then, the absorbance of the solution was measured at 230 nm. The HPSA was calculated using the following formula:

HPSA (%) = [(*c* − *c_b_*) − (*s* − *s_b_*)]/(*c* − *c_b_*) × 100% (1)

where *c* is absorbance of the H_2_O_2_ with the PBS, *c_b_* is absorbance of the PBS, *s* is absorbance of the sample and the H_2_O_2_ with the PBS and *s_b_* is absorbance of the sample and the PBS. Ferulic acid dissolved in methanol was used to prepare the standard curve, the HPSA was expressed as μmol of FAE per gram of dry sample (μmol FAE/g DS).

### 3.7. Determination of DPPH Radical Scavenging Activity (DRSA)

The determination of the effect of extracts on DRSA was based on a procedure as determined by Hatano et al. [45] and Villanueva-Carvajal et al. [43], with slight modifications. Briefly, 1 mL of the phenolic extract was mixed with 4 mL 79 μmol/L methanolic DPPH solution and shaken vigorously. Absorbance was measured at 517 nm after the solution was incubated in the dark at ambient temperature for 10 min. The radical scavenging activity was calculated using the following formula:

DRSA (%) = [(*c* − *c_b_*) − (*s* − *s_b_*)] / (*c* − *c_b_*) × 100% (2)

where *c* is absorbance of the DPPH solution, *c_b_* is absorbance of the methanol, *s* is absorbance of the DPPH solution with the sample, and *s_b_* is absorbance of the methanol with the sample. The standard curve was prepared using ferulic acid and expressed as μmol of FAE per gram of dry sample (μmol FAE/g DS).

### 3.8. Determination of Trolox Equivalent Antioxidant Capacity (TEAC)

The TEAC of the extracts was determined as described by Re et al. [46], with slight modifications. Briefly, 100 µL of phenolic extract was mixed in 3.8 mL ABTS working solution (7 mmol/L ABTS mixed with 2.45 mmol/L potassium persulfate in a volume ratio of 1:1). The absorbance at 734 nm was measured in the dark for 6 min. The TEAC was calculated using the following formula:

TEAC (%) = [(*c* − *c_b_*) − (*s* − *s_b_*)]/(*c* − *c_b_*) × 100% (3)

where *c* is absorbance of the ABTS working solution, *c_b_* is absorbance of the ethanol, *s* is absorbance of the sample with the ABTS working solution, and *s_b_* is absorbance of the sample with the ethanol. Trolox was used as a reference standard, and the TEAC was expressed as μmol of Trolox equivalents (TE) per gram of dry sample (μmol TE/g DS).

### 3.9. HPLC Analysis

All the phenolic fractions were injected into a high-performance liquid chromatography (HPLC) system (Agilent Technologies, Palo Alto, CA, USA) equipped with a G1315B diode array detector (DAD) and a G1316A column compartment. The separation was performed on a 150 mm × 4.6 mm, 5μm Agilent Zorbax SB-C18 at 30 °C. Its system controller was linked to a ChemStation for LC 3D systems (Agilent Technologies). The mobile phase consisted of methanol (Solvent A) and water with 0.5% formic acid (Solvent B). The flow rate was maintained at 0.8 mL/min. The gradient program was as follows: 0 min, A:B (5:95, *v*/*v*); 20 min, A:B (95:5, *v*/*v*); 21 min, A:B (5:95, *v*/*v*); and 25 min, A:B (5:95, *v*/*v*). The detect wavelength was set at 280 nm. Identification and quantification of the 18 phenolic compounds were based on the RT and characteristic absorption spectrum from the DAD with those of their authentic standards. The quantitation of each phenolic compound was carried out using an external standard method. Available pure known compounds as external standards were used for quantifying samples.

### 3.10. Statistical Analysis

All the analyses were performed, and the results were expressed as the mean ± the standard deviation of three replicates. An independent-sample *t*-test was performed to determine differences between the two kinds of CM extracts at *p* < 0.05. Statistical analysis was undertaken using SPSS version 22.0 software (SPSS Inc., Chicago, IL, USA).

## 4. Conclusions

The phenolic compounds and antioxidant activities of phenolic extracts in CMP were firstly studied. Moreover, the content, antioxidant activities and phenolic compounds of soluble and insoluble-bound phenolic extracts in CMC and CMP were also compared. The results of this study showed that the TPC and TFC of soluble phenolic extracts were higher than insoluble-bound phenolic extracts in both CMC and CMP. In addition, the TPC of soluble, insoluble-bound and total phenolic compounds in CMP were significantly higher than those in CMC (*p* < 0.05). The soluble phenolic extracts in CMP showed higher antioxidant activities than those in CMC. Eighteen phenolic compounds from phenolic extracts in these two CMs were identified and quantified by HPLC, respectively. Vanillic acid (1325.77 ± 23.34 μg/g) and myricetin (2209.79 ± 1476.96 μg/g) might be characteristic phenolic compounds in soluble phenolic extracts of CMC and CMP. The phenolic compounds of these two CMs represent a potential source of antioxidants. Therefore, CMC and CMP, especially the latter, may play an important role in human health. It is necessary to explore and possibly promote their use as functional food additives.

## Figures and Tables

**Table 1:** Antioxidant activities of the two Chinese mistletoes.

Plant Material	CMC	CMP
**Ferric Reducing Antioxidant Power (μmol FE/g DS)**		
Soluble	42.25 ± 1.49a ^2^	44.76 ± 0.32a ^1^
Insoluble-bound	8.07 ± 0.75b ^2^	10.31 ± 0.46b ^1^
**Hydrogen Peroxide Scavenging Activity (μmol FAE/g DS)**		
Soluble	1429.34 ± 7.69a ^1^	1431.87 ± 4.16a ^1^
Insoluble-bound	1383.79 ± 3.33b ^1^	1231.67 ± 12.23b ^2^
**DPPH Radical Scavenging Activity (μmol FAE/g DS)**		
Soluble	2.19 ± 0.11a ^2^	2.51 ± 0.04a ^1^
Insoluble-bound	1.51 ± 0.07b ^2^	1.83 ± 0.09b ^1^
**Trolox Equivalent Antioxidant Capacity (μmol TE/g DS)**		
Soluble	81.03 ± 0.90a ^2^	84.92 ± 1.50a ^1^
Insoluble-bound	5.78 ± 1.24b ^1^	1.40 ± 0.24b ^2,^*

* CMC, the Chinese mistletoes hosted by *Camellia assamica* (Mast.) Chang; CMP, the Chinese mistletoes hosted by *Pyrus*, *i*, *f.*; FE, Fe^2+^ equivalents; FAE, ferulic acid equivalents; TE, Trolox equivalents; DS, dry sample. Values are mean ± standard deviation (*n* = 5); Values in each row having the different superscripts are significantly different (*p* < 0.05); values in each column having the different letter values are significantly different (*p* < 0.05).

**Table 2:** Individual soluble phenolic compounds in the two Chinese mistletoes (μg/g DS sample).

Phenolic Compounds	CMC	CMP
**Hydroxybenzoic Acids**		
Gallic acid	67.51 ± 12.21	172.65 ± 4.34
Protocatechuic acid	39.65 ± 9.27	203.23 ± 12.89
*p*-Hydroxybenzoic acid	133.88 ± 116.31	787.95 ± 138.89
Vanillic acid	1325.77 ± 23.34	408.82 ± 29.21
Syringic acid	432.33 ± 370.24	66.68 ± 27.90
Vanillin	70.08 ± 36.40	136.92 ± 130.03
Total	2069.23	1776.25
**Hydroxycinnamic Acids**		
Chlorogenic acid	93.36 ± 50.71	68.85 ± 27.70
Caffeic acid	105.59 ± 12.03	119.18 ± 16.77
*p*-Coumaric acid	85.05 ± 70.36	820.10 ± 355.68
Ferulic acid	560.48 ± 25.71	248.82 ± 101.63
*trans*-Cinnamic acid	67.80 ± 9.00	172.60 ± 1.93
Total	912.28	1429.55
**Flavonoids**		
Catechin hydrate	188.60 ± 138.25	200.51 ± 150.87
Epicatechin	238.18 ± 79.30	569.53 ± 67.92
(−)-Epigallocatechin	237.03 ± 8.36	542.10 ± 23.71
Myricetin	154.88 ± 62.10	2209.79 ± 1476.96
Quercetin	85.22 ± 28.75	834.71 ± 543.19
Kaemferol	40.48 ± 10.23	197.63 ± 157.76
Apigenin	25.50 ± 5.66	447.18 ± 3.32
Total	969.89	5001.44 *

* CMC: the Chinese mistletoes hosted by *Camellia assamica* (Mast.) Chang; CMP: the Chinese mistletoes hosted by *Pyrus*, *i*, *f.*; Values are mean ± standard deviation (*n* = 3).

**Table 3:** Individual insoluble-bound phenolic compounds in the two Chinese mistletoes (μg/g DS sample).

Phenolic Compounds	CMC	CMP
**Hydroxybenzoic Acids**		
Gallic acid	6.34 ± 0.56	21.41 ± 0.92
Protocatechuic acid	27.43 ± 1.73	23.24 ± 4.45
*p*-Hydroxybenzoic acid	48.02 ± 22.44	55.20 ± 42.12
Vanillic acid	52.73 ± 8.40	37.40 ± 25.69
Syringic acid	6.39 ± 1.14	10.45 ± 6.34
Vanillin	13.18 ± 7.35	39.69 ± 2.06
Total	154.08	187.40
**Hydroxycinnamic Acids**		
Chlorogenic acid	12.28 ± 2.43	22.21 ± 12.98
Caffeic acid	49.88 ± 2.41	28.20 ± 1.19
*p*-Coumaric acid	206.97 ± 21.39	14.26 ± 13.13
Ferulic acid	97.94 ± 4.63	171.18 ± 4.88
*trans*-Cinnamic acid	43.06 ± 1.02	124.38 ± 2.31
Total	410.12	360.23
**Flavonoids**		
Catechin hydrate	129.17 ± 32.41	92.21 ± 2.78
Epicatechin	11.21 ± 9.76	26.34 ± 12.67
(−)-Epigallocatechin	14.63 ± 10.29	223.32 ± 24.87
Myricetin	33.14 ± 19.55	75.23 ± 49.31
Quercetin	41.44 ± 11.71	62.30 ± 31.66
Kaemferol	18.15 ± 12.05	99.40 ± 69.46
Apigenin	10.35 ± 9.34	9.33 ± 6.64
Total	258.10	588.13 *

* CMC: the Chinese mistletoes hosted by *Camellia assamica* (Mast.) Chang; CMP: the Chinese mistletoes hosted by *Pyrus*, *i*, *f*.; Values are mean ± standard deviation (*n* = 3).

## Supplementary Materials

Supplementary materials are available online. Figure S1. HPLC chromatograms of 18 phenolic standard samples.
