# Supplementary figures and images for: Comparative Evaluation of Soluble and Insoluble-Bound Phenolics and Antioxidant Activity of Two Chinese Mistletoes
Source: Molecules. 2018 Feb 8;23(2):359. doi: 10.3390/molecules23020359 (PMC6017001; doi:10.3390/molecules23020359)

# 1 Supplementary Material

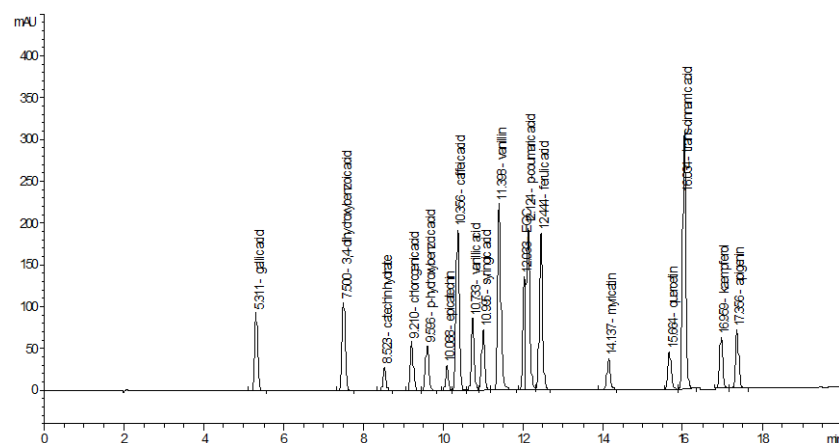

**Figure 1S.** HPLC chromatograms of 18 phenolic standard samples.

Supplement: Supplementary file 1 [file molecules-23-00359-s001.pdf]
